# Case Report: preoperative prediction of aneurysm rupture site using aortic morphological and biomechanical analysis validated by intraoperative imaging

**DOI:** 10.3389/fcvm.2025.1629547

**Published:** 2025-08-21

**Authors:** Tianming Huang, Yifeng Pan, Shuangxiang Lin, Yuanming Luo, Bing Chen

**Affiliations:** ^1^ State Key Laboratory of Transvascular Implantation Devices, the Second Affiliated Hospital, Zhejiang University School of Medicine, Hangzhou, China; ^2^ Department of Technology, Boea Wisdom (Hangzhou) Network Technology Co., Ltd., Hangzhou, China; ^3^ Department of Vascular Surgery, The Second Affiliated Hospital, Zhejiang University School of Medicine, Hangzhou, China; ^4^ Department of Radiology, The Second Affiliated Hospital, Zhejiang University School of Medicine, Hangzhou, China; ^5^ Department of Mechanical Engineering, The University of Iowa, Iowa City, IA, United States

**Keywords:** aneurysm rupture prediction, biomechanical analysis, multidimensional dynamic CTA, multimodal imaging, abdominal aortic aneurysm

## Abstract

Prediction of aneurysm rupture has been a great challenge for decades. We report a successful rupture site prediction on a 97 mm abdominal aortic aneurysm (AAA). A 73-year-old man with an 11-year history of AAA presented to our outpatient clinic with a one-week history of hemoptysis. After undergoing multidimensional dynamic CTA imaging, the high rupture risk region was predicted through comprehensively inspecting the image-derived characteristics of biomechanics, morphology, and intraluminal thrombus distribution. Owing to financial difficulties, the patient declined treatment. The patient returned to the hospital 140 days later with severe acute abdominal pain. Follow-up CT imaging revealed contrast extravasation accompanied by a large retroperitoneal hematoma, indicating active aneurysmal rupture. Emergency endovascular aneurysm repair (EVAR) was subsequently performed. Notably, the rupture site corresponded to the region previously predicted by our biomechanical analysis and was confirmed intraoperatively via digital subtraction angiography. The patient's postoperative course was uneventful, and he remained in stable condition at the 3-month follow-up. This successful prediction serves as a starting point for verifying the considerations regarding aneurysm rupture mechanism, which would benefits innovative treatment options in future.

## Introduction

Abdominal aortic aneurysm (AAA) is often referred to as a “silent killer” due to its asymptomatic nature, and its rupture is a life-threatening acute event. A 2022 meta-analysis found an 81% fatality rate overall after rupture (including 1/3 of patients who die before reaching any hospital) (1). Currently, the clinical challenge is precise decision of timing for treatment when an aneurysm is found. The guidelines suggest treatment when maximal aneurysm diameter exceeds 55 mm (2, 3). However, this criterion fails to prevent rupture in 10%–24% of ruptured AAAs (4, 5). Additionally, unruptured AAAs larger than 55 mm are frequently encountered in clinical practice. Various factors have been explored to make more accurate evaluation about rupture risk, such as intraluminal thrombus (ILT) (6–8), geometry (9), smoking (10), biomechanical characteristics (11, 12), et al.

Computed tomography angiography (CTA) is an important and most prevailing imaging technique for capturing aneurysm geometry. The electrocardiographic-gating (ECG) technique extends the capability of CTA for tracking vascular boundary deformation during cardiac cycles, which is also called ECG multidimensional dynamic CTA (MD-CTA). Two MD-CTA based new indicators *SSI* and *dSSI* were recently proposed based on *in vivo* biomechanical analysis, which provide more patient-specific evaluation on the mechanical conditions of aneurysms and supplement the diameter criterion (13).

Despite significant advances in imaging and biomechanical modeling, real-world evidence validating patient-specific predictions of AAA rupture location remains extremely limited. This gap exists largely because most large aneurysms are treated preemptively, while ruptures often occur unpredictably and leave insufficient time for detailed analysis. Here, we present a rare case of a 97 mm AAA in which the region at highest risk of rupture was preoperatively identified through a combination of MD-CTA-derived biomechanical parameters–including strain, wall tension, *SSI* (Scaled Stiffness Index), and *dSSI* (Scaled Stiffness Hardening Index)–together with morphological and ILT characteristics. The patient declined elective repair and returned four months later with aneurysm rupture. Intraoperative digital subtraction angiography (DSA) confirmed that the rupture occurred in the previously predicted high-risk region. This case uniquely demonstrates the potential clinical utility of regional, image-based mechanical assessment for accurate rupture prediction, providing compelling real-world support for a more nuanced, patient-specific approach to AAA management.

## Case description

**Figure 1:**
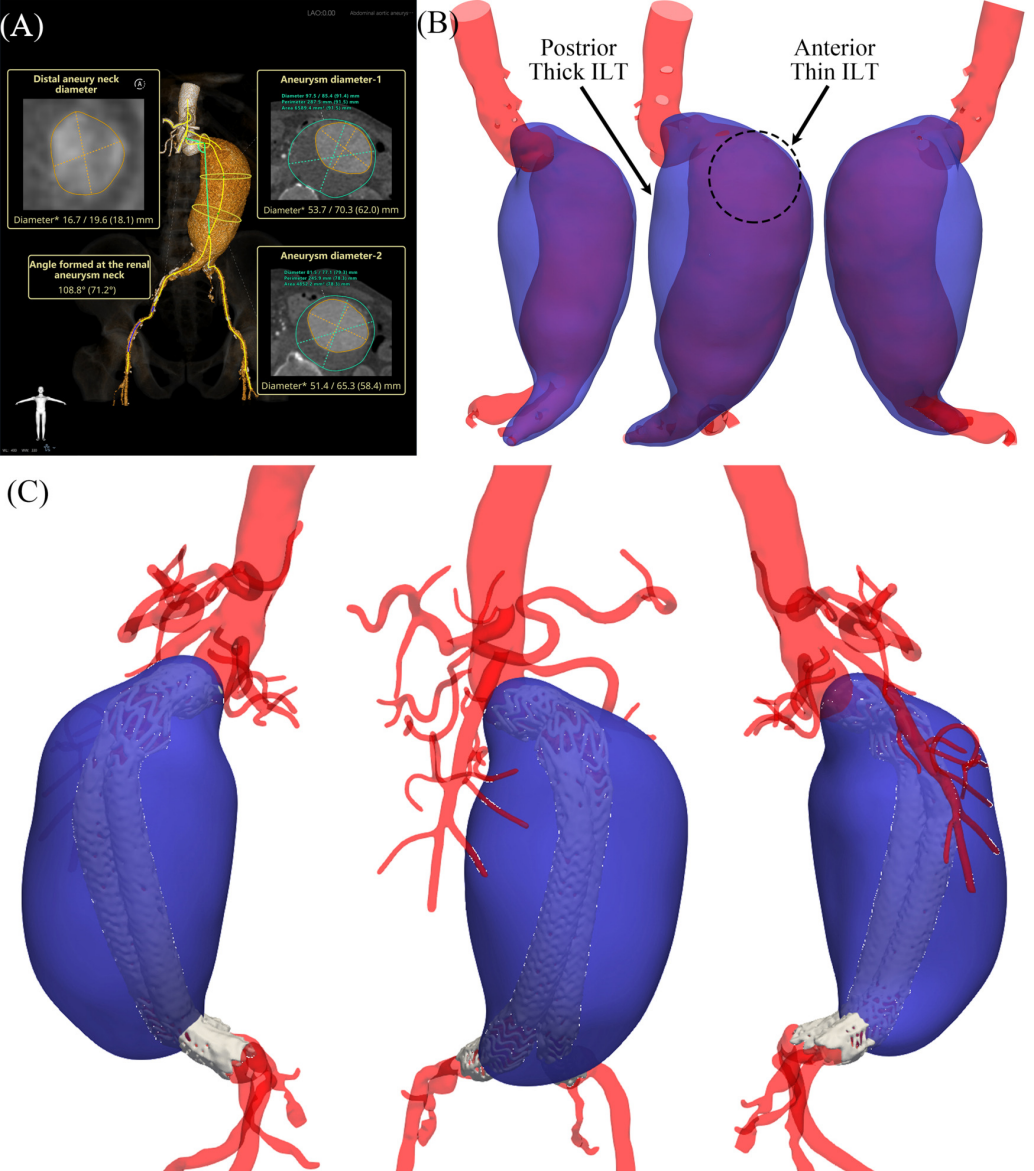
Morphological results. **(A)** Measurement report; **(B)** aneurysm and intraluminal thrombus models reconstructed from preoperative images; **(C)** models reconstructed from postoperative images.

**Figure 2:**
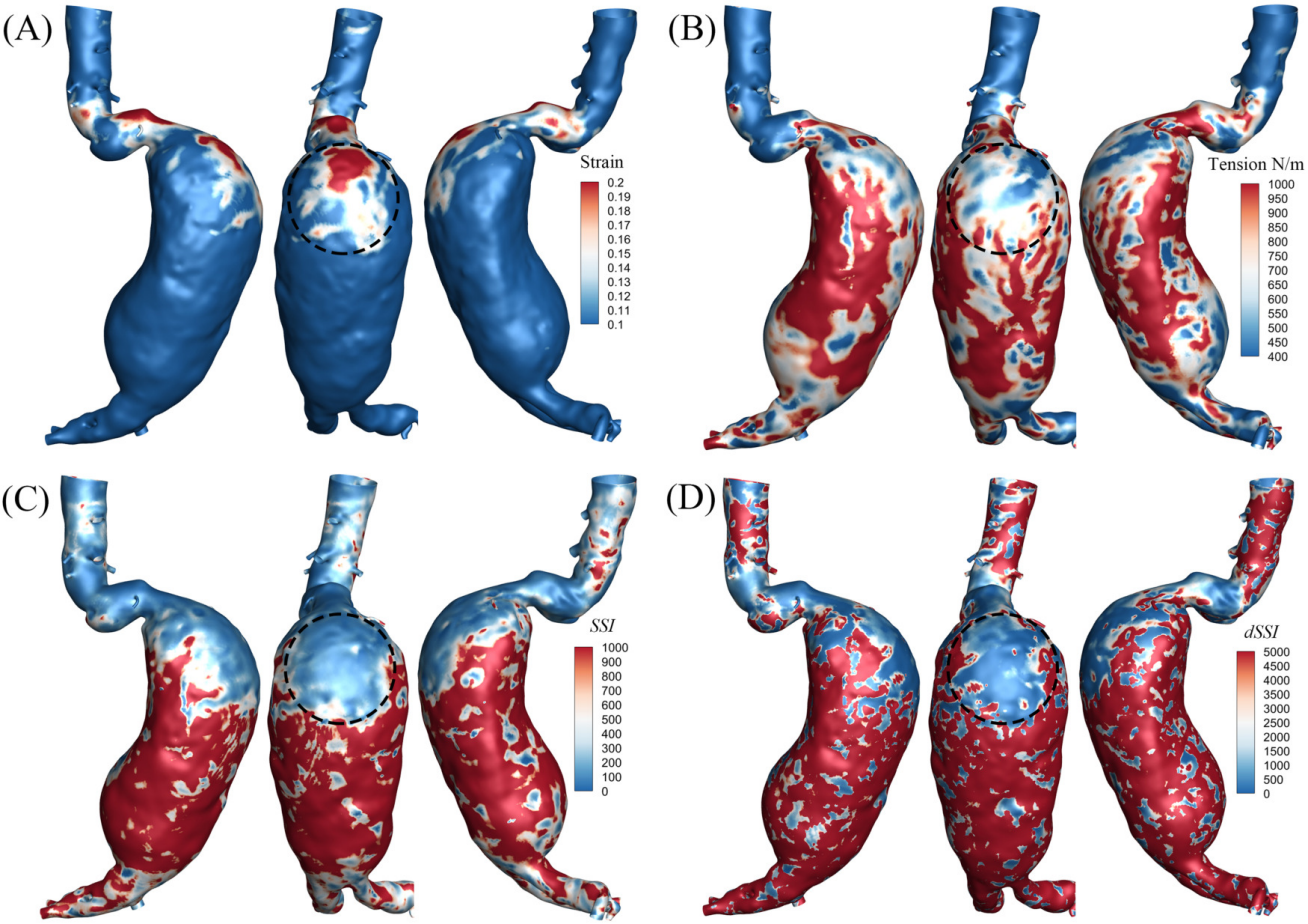
Results of mechanical characteristics. **(A)** Distribution of strain, average strain on the aneurysm body is 0.058, and average strain within the marked region exceeds 0.1; **(B)** distribution of tension, average wall tension on the aneurysm body is 9.7E2 N/m, and average wall tension within the marked region is 6.2E2 N/m; **(C)** distribution of SSI, average SSI on the aneurysm body is 7.8E2, and average SSI within the marked region is 1.2E2; **(D)** distribution of dSSI, average dSSI on the aneurysm body is 2.2E4, and average dSSI within the marked region is 1.6E3.

On 7 Mar. 2023, a 73-year-old man with an 11-year history of AAA presented to our outpatient clinic with a one-week history of hemoptysis (Table 1). His medical history mainly included hypertension and rectal cancer status post-surgical resection. On 8 Mar., the patient underwent MD CTA imaging (see Supplementary Video S1), and his blood pressure was 168/94 mmHg at the time of scanning. The maximal aneurysm diameter was up to 97 mm through measurement, as shown in Figure 1A. The patient was clinically considered at high risk. With the approval of Ethics Committee of the Second Affiliated Hospital Zhejiang University School of Medicine, more numerical analysis based on the collected MD CTA images then followed. The aneurysm and ILT models, as shown in Figure 1B, were annotated and reconstructed with Insight Toolkit (ITK). The mechanical characteristics, including lumen strain, tension, *SSI* and *dSSI*, were identified based on the collected MD-CTA images and presented in Figure 2. The biomechanical indicators shown in Figure 2 were derived using a previously established method based on MD-CTA imaging (13). In brief, aortic lumen geometries at multiple cardiac phases were extracted and registered using a non-rigid diffeomorphic algorithm to compute voxel-level displacement. A surrogate membrane model was then constructed by extruding the lumen surface with uniform thickness, and inverse finite element analysis was performed to estimate lumen tension under patient-specific blood pressures. Regional strain and tension were used to fit nonlinear tension–strain curves, from which two mechanical indices *SSI* and *dSSI*, were computed to reflect aortic stiffness and strain-hardening behavior. The average value of *SSI* on the aneurysm body exceeds 700.0, indicating that the aneurysm was highly stiffened compared to the median value of the controls reported in the previous work (13). The average *dSSI* on the aneurysm body exceeds 20,000.0, which is also significantly higher than the median value of the controls. Considering the aneurysm's diameter, morphological features, ILT, and biomechanical characteristics, immediate elective interventional repair was strongly recommended for him. Owing to financial difficulties, the the patient declined the procedure and was discharged on 9 March.

**Table 1:** Patient timeline.

Timeline	Diagnosis and treatment
2012	• Was found to have an abdominal aortic aneurysm.
07/Mar./2023	• Presented to the outpatient clinic with a one-week history of hemoptysis. • Admitted to the hospital.
08/Mar./2023	• Multidimensional CTA scanning. • Analysis of morphological and wall mechanical characteristics and intraluminal thrombus distribution.
09/Mar./2023	• Elective surgical repair recommendation.
10/Mar./2023	• Patient declined surgical intervention due to financial concerns and left the hospital.
26/Jul./2023	• The patient returned with acute abdominal pain, admitted to the hospital. • Emergency CTA indicated suspicion of aneurysm rupture.
27/Jul./2023	• The patient underwent emergency endovascular aneurysm repair (EVAR). • Intraoperative DSA confirmed that the rupture site.
16/Aug./2023	• Discharged after successful treatment.
13/Nov./2023	• Stabilized following EVAR at the 3-month follow-up.

The region marked in Figure 2 was predicted with high rupture risk based on the following consideration: (i) high strain response concentratedly distributed in this region, the average value of strain on the aneurysm body is 0.058, the average strain within the marked region exceeds 0.1 which means its boundary with the adjacent surrounding low strain region contains a steep gradient boundary, as shown in Figure 2A; (ii) low wall tension response as well concentratedly distributed in this region, as shown in Figure 2B; (iii) both *SSI* and *dSSI* contain a steep gradient at the boundary with its adjacent surrounding region, as shown in Figures 2C,D; (iv) the ILT in this region is relatively thin, as shown in Figure 1B.

**Figure 3:**
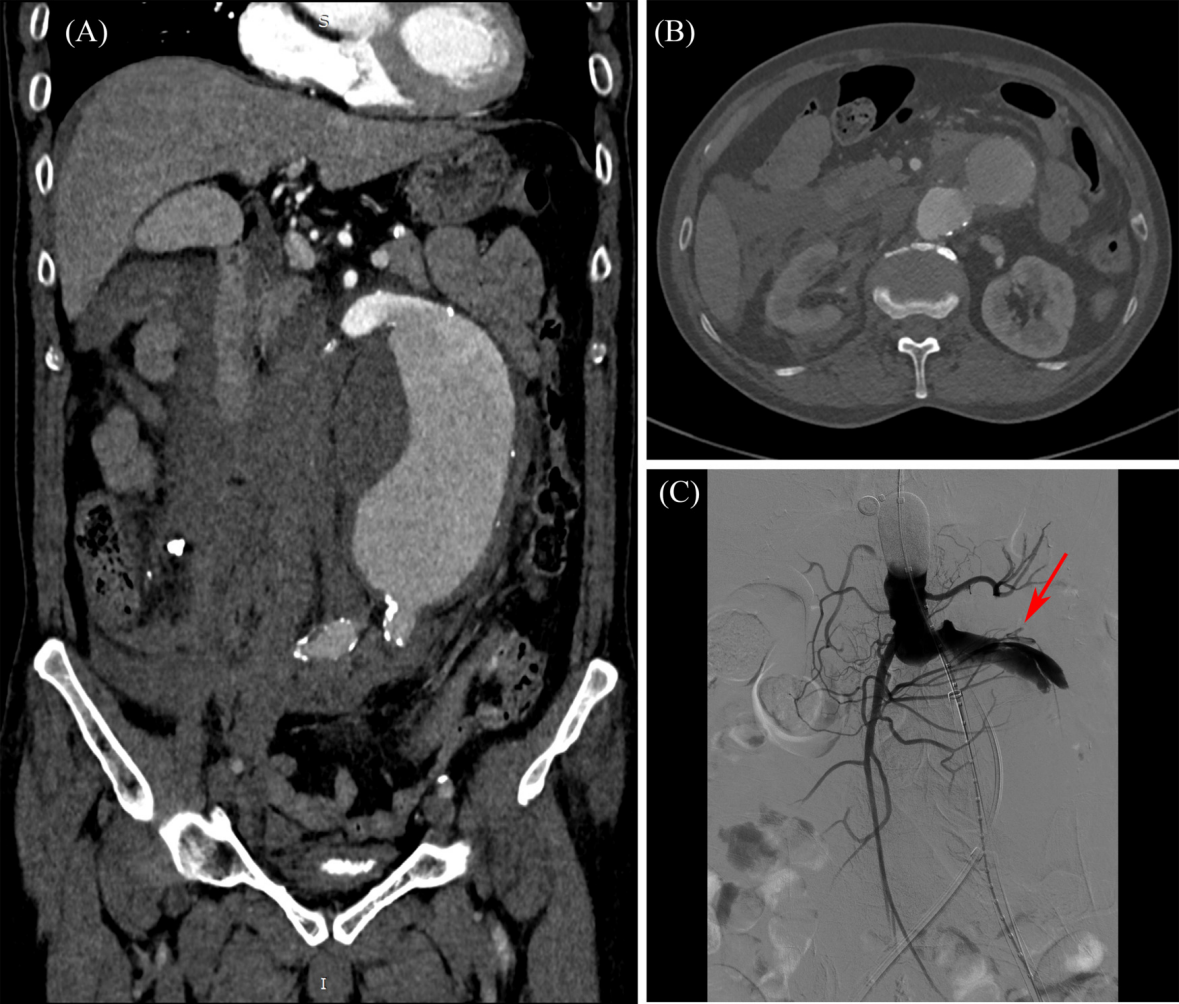
Preoperative and intraoperative imaging of the abdominal aortic aneurysm. **(A)** Preoperative CTA–coronal view; **(B)** preoperative CTA–cross-sectional view at aneurysm neck level; **(C)** intraoperative DSA–Rupture site captured by DSA imaging.

On 26 Jul. 2023, 140 days later, the patient came back to the hospital with severe acute abdominal pain, and was admitted immediately. The patient was scheduled to undergo endovascular aneurysm repair (EVAR) with a covered stent graft following the completion of necessary preoperative evaluations, including abdominal CTA. The blood pressure dropped down to 76/44 mmHg in hospital day 2, and an emergency CT scan was performed. Contrast extravasation with a large retroperitoneal hematoma, as illustrated in Figures 3A,B, was observed comparing to the observation of hospital day 1, and aneurysm rupture was suspected. Emergency interventional procedure followed. A GORE® RLT covered stent graft (31 × 14 × 170 mm) was deployed below the renal arteries. The left limb was extended with a GORE® iliac covered stent graft (16 × 20 × 140 mm), and the right limb was extended with another GORE® iliac covered stent graft (16 × 23 × 140 mm). The digital subtraction angiography (DSA) images (see Supplementary Video S2), recorded during the interventional surgery, are presented in Figure 3C. The rupture site is indicated by red arrow, which locates in the predicted high rupture risk region. The patient was discharged on August 16 following successful treatment and remained clinically stable at the 3-month follow-up visit on November 13, as illustrated in Figure 1C and Supplementary Video S3. At that time, the patient reported no abdominal pain or discomfort and expressed overall satisfaction with the recovery process. No new symptoms or complications were observed.

## Discussion

The mortality rate following AAA rupture approaches 100% in the absence of emergency intervention (5, 14). Given that most AAAs remain asymptomatic until rupture, non-invasive detection and patient-specific risk evaluation are of critical importance. A CTA scan is one of the most prevailing examinations for non-invasive detection, and the remaining challenge now is how to non-invasively evaluate patient-specific rupture risk for a detected aneurysm. Randomized controlled trials suggest that early repair for small AAA (40 mm to 55 mm) does not reduce mortality (15). For large aneurysms, the rupture risk is 2.2% and 6.0% over 3 years for 55–60 mm and 61–70 mm AAA, respectively (16). For most patients undergoing elective open surgical repair (OSR) or endovascular aneurysm repair (EVAR), the clinical significance is to prevent final rupture which leads to mortal adverse events. A recent study reported that the 3-year cumulative incidence of rupture was 18.4% even for huge AAA (>70 mm) (16). The commonly used 55 mm is a relatively conservative criterion, which is proposed to a great extent due to the lack of effective methods for *in vivo* non-invasive patient-specific rupture risk assessment. An effective and patient-specific evaluation method can help more precisely manage aneurysms, that is to say, to reduce unexpected ruptures of small AAAs and unnecessary elective repairs of large AAAs. This is of great significance and remains a research priority.

The biomechanical related researches are increasingly reported in an attempt to enrich the factors affecting rupture (11, 17, 18). Nevertheless, few outcomes are yet qualified to be applied. Tracking the deformation of lumen wall during cardiac cycles through MD CTA provides a new opportunity to make patient-specific assessment on the mechanical status of aneurysms (13). The mechanical characteristics, lumen strain, tension, *SSI* and *dSSI* are all non-invasively *in vivo* identified (13). The *SSI* and *dSSI* are demonstrated to be significantly correlated to the current criterion diameter but reveal more patient-specific and regional quantitative evaluation. However, real world evidence is still lacking.

The valuable case reported here provides an opportunity to verify our prediction from multi-point of view. From the perspective of biomechanics, concentration of mechanical quantities generally accompany higher rupture risk. The general strain range in healthy aortas is around 0.2 (13, 19). The overall strain or deformability of this AAA is relatively low. However, as shown in Figure 2A, the strain distribution presents high concentration in the marked region on the AAA belly. By contrast with this concentrated high value region (>0.1), the strain of the adjacent surrounding region on the aneurysm body is even much lower (0.058 for average). The boundary between the high strain region and the surrounding low strain region forms a concentrated non-smoothing transition, or steep strain gradient boundary. The generally range of aortic tension for healthy individuals is around 50–200 N/m (13, 19). The average tension of this AAA is up to 1,000. Similarly, there is also a concentrated low tension region, as shown in Figure 2B. The steep gradient boundary existing in the distribution of strain and tension is generally consistent, i.e., the marked region. Such steep gradient boundary is considered to contain higher risk. This is the first factor of consideration to predict the rupture region.

*SSI* and *dSSI* distributions both present steep gradient on the consistent boundary presented in the strain and tension distribution. *SSI* and *dSSI* are quantities reflecting the material properties of aneurysm wall. The generally range of *SSI* for healthy individuals varies from single digits to several tens (13, 19). In contrast, patients with aneurysms frequently exhibit values exceeding 100, and in some cases, reaching several hundreds. Averaged SSI of this case is up to 700, with localized regions surpassing 1,000, which is substantially higher than typical values observed in common aneurysms. *dSSI* exhibited a similar trend. The steep gradient of material characteristics is an important factor resulting in the concentration of mechanical quantities. The *SSI* and *dSSI* are significant lower in the marked region than the surrounding outer region, as shown in Figures 2C,D. This also explains that the middle and lower region of the aneurysm belly is much more stiffened than the marked region. This also explains that the strain and tension present the features as shown in Figures 2A,B. The steep gradient of the material stiffness characteristics is the second factor of consideration to predict the rupture region.

In the context of AAA, the presence of ILT is almost inevitable, as approximately 75% of AAAs contain ILT of varying extent (7, 20, 21), and this case is no exception, as shown in Figure 1B. The influence of ILT on aneurysm progression and rupture remains controversial, with some studies suggesting a protective role and others indicating adverse effects (20, 22–24). Prior research has shown that aneurysms tend to rupture more easily on the anterior wall, potentially due to better oxygenation and nutrient supply of the posterior wall via the lumbar arteries (25). Moreover, thinner ILT regions are generally considered more vulnerable than thicker ones (26, 27). In this case, as shown in the annotated Figure 1B, the ILT on the upper anterior wall of the aneurysm is noticeably thinner than that on the posterior side. In this case, as shown in the annotated Figure 1B, the ILT on the upper anterior wall of the aneurysm is noticeably thinner than that on the posterior side. The abnormal geometry of the aneurysm neck appears to direct blood flow more strongly toward this anterior region, reducing local thrombus formation. In contrast, the posterior wall exhibits a significantly thicker ILT layer. These morphological and hemodynamic features suggest that the anterior region—characterized by thinner ILT and greater flow impact—is more susceptible to rupture. This observation supports the localization of the rupture-prone region highlighted in our analysis.

Although clinical interventions such as OSR and EVAR typically address the entire aneurysm, the value of regional rupture risk assessment should not be underestimated. It is paradoxical to accurately predict rupture risk without considering regional variations within the aneurysm. Localized mechanical weaknesses may explain why some small aneurysms rupture unexpectedly, despite low overall risk based on diameter alone. Furthermore, as ongoing research continues to yield new treatments, such as the natural vascular scaffold (NVS), regional assessment is poised to play an increasingly significant role in the development of innovative therapeutic strategies. Interventional treatment techniques are striving for fewer implants, and regional assessment is critical for more localized treatment strategies.

It is important to note that more matched regions may appear if only a single risk factor is considered. For instance, the neck region also exhibits high strain and contains a steep gradient boundary, while the middle anterior belly of the aneurysm is characterized by a thin ILT. However, only the marked region aligns all high risk factors. Significant efforts have been dedicated to predicting rupture risk. Yet, no reliable indices or methods have been established for clinical application. Aneurysm rupture is a highly complex event influenced by multiple factors. While the evidence presented here is logical and internally consistent, further cases are needed to validate the reliability of this predictive approach.

## Conclusion

In conclusion, we made a successful prediction on the rupture region of an AAA case by comprehensively analyzing image-derived biomechanics indicators, ILT distribution and morphology. This work advances patient-specific regional risk assessment of aneurysm rupture in a practical way, and may support the development of more precise and minimally invasive treatment strategies.

## Patient perspective

I was told my aneurysm had a high risk of rupture, but I delayed treatment due to financial concerns. Months later, it ruptured exactly where the doctors had predicted, and I'm grateful their quick intervention saved my life.

## Data Availability

The raw data supporting the conclusions of this article will be made available by the authors, without undue reservation.

## Supplementary material

Supplementary Vedio 1 Preoperative CTA image

Supplementary Vedio 2 Intraoperative DSA

Supplementary Vedio 3 Postoperative CTA image
